# Zika Virus Exploits Lipid Rafts to Infect Host Cells

**DOI:** 10.3390/v14092059

**Published:** 2022-09-16

**Authors:** Daniela Peruzzu, Antonello Amendola, Giulietta Venturi, Valeria de Turris, Giulia Marsili, Claudia Fortuna, Katia Fecchi, Maria Cristina Gagliardi

**Affiliations:** 1 Center for Gender Specific Medicine, Istituto Superiore di Sanità, Viale Regina Elena 299, 00161 Rome, Italy; 2 Department of Infectious Diseases, Istituto Superiore di Sanità, Viale Regina Elena 299, 00161 Rome, Italy; 3 Istituto Italiano di Tecnologia, CLN2S-Nanotechnology for Neuroscience, Viale Regina Elena 291, 00161 Rome, Italy

**Keywords:** lipid rafts, flavivirus, zika virus, ampothericin B, virus-cell interaction

## Abstract

Several flaviviruses such as Hepatitis C virus, West Nile virus, Dengue virus and Japanese Encephalitis virus exploit the raft platform to enter host cells whereas the involvement of lipid rafts in Zika virus–host cell interaction has not yet been demonstrated. Zika virus disease is caused by a flavivirus transmitted by *Aedes* spp. Mosquitoes, although other mechanisms such as blood transfusion, sexual and maternal–fetal transmission have been demonstrated. Symptoms are generally mild, such as fever, rash, joint pain and conjunctivitis, but neurological complications, including Guillain-Barré syndrome, have been associated to this viral infection. During pregnancy, it can cause microcephaly and other congenital abnormalities in the fetus, as well as pregnancy complications, representing a serious health threat. In this study, we show for the first time that Zika virus employs cell membrane lipid rafts as a portal of entry into Vero cells. We previously demonstrated that the antifungal drug Amphotericin B (AmphB) hampers a microbe–host cell interaction through the disruption of lipid raft architecture. Here, we found that Amphotericin B by the same mechanism of action inhibits both Zika virus cell entry and replication. These data encourage further studies on the off-label use of Amphotericin B in Zika virus infections as a new and alternate antiviral therapy.

## 1. Introduction

Several studies revealed that different microbial pathogens such as viruses, bacteria and protozoa employ host cell lipid rafts as cell surface platform to interact, bind and possibly enter into host cells [1,2]. Lipid rafts are historically proposed as small, highly dynamic plasma membrane microdomains enriched in cholesterol, glycosphingolipids and phospholipids. These membrane regions play an important role in a variety of cellular functions, but principally they recruit and concentrate several molecules and receptors involved in cellular signaling, forming a sort of signal transduction platform [3]. Lipid rafts also cluster several pathogen recognition receptors into a “phagocytic synapse” and consequently are the focus of intense research in the field of infection. They are involved at different steps of host–microbe interaction, starting from adhesion/binding, establishment and maintenance of the vacuole and microbe activation of signaling pathways [4].

For viruses, host cell lipid rafts have been reported to be involved in several steps of viral entry and also in the late processes of assembly, budding and release of viral particles. They may act as a platform to concentrate virus receptors or virus proteins involved in virion assembly and they also address the virus to the right intracellular sites. The role of membrane rafts has been ascertained in the life cycle of several both enveloped and non-enveloped, DNA and RNA viruses and confirmed by the antiviral effects of raft-disrupting agents on infection and viral replication [5].

Flaviviruses are enveloped, single-stranded RNA viruses comprising several important human pathogens, such as Hepatitis C virus (HCV), West Nile virus (WNV), Dengue virus (DENV), Japanese encephalitis virus and Zika virus (ZIKV) [6]. ZIKV was discovered in Africa in 1947 and has circulated through Africa and Asia as an under-noticed agent causing either asymptomatic infections or self-resolving influenza-like illness [7]. In 2007, the first ZIKV outbreak was reported from the Island of Yap (Federated States of Micronesia). This was followed by a large outbreak of ZIKV virus infection in French Polynesia in 2013, and, starting from March 2015, in Brazil [8]. These large outbreaks revealed that ZIKV can cause serious neurological diseases, such as Guillain-Barré syndrome in adults, a polyneuropathy mediated by the immune system that can lead to paralysis and death [9] and microcephaly in newborns. *Aedes* spp. are the major vectors for horizontal transmission of ZIKV to humans, although other non-vector-borne mechanisms of transmission, such as blood transfusion, sexual and maternal–fetal transmission, have been demonstrated for this virus [10]. Foetuses infected with the virus during pregnancy may develop a range of pathologies including microcephaly, cerebral calcifications and macular scarring, collectively known as Congenital Zika syndrome [11].

Lipid rafts are involved in different steps of viral entry and life cycle of several flaviviruses such as DENV, HCV, WNV [12], but the involvement of lipid rafts in ZIKV-host cell interaction has not yet been demonstrated.

The aim of our work was to investigate whether ZIKV could exploit lipid rafts to enter and infect host cells in order to identify a putative therapeutic target.

## 2. Materials and Methods

### 2.1. Cells, Virus and Drug

African green monkey Vero cells were grown in MEM 1× + Glutamax (Gibco, Billings, MT, USA) supplemented with 10% foetal bovine serum, antibiotics and aminoacids (Gibco, Billings, MT, USA). ZIKV H/PF/2013 strain of the Asian genotype (Baronti et al., 2014) was grown in Vero cells and titrated by plaque assay. AmphB and Methyl-β-Cyclodextrin (MβCD) were purchased by Gibco and Sigma-Aldrich (St. Louis, MO, USA) respectively.

### 2.2. Virus Infection and Drug Treatments

Vero cells were cultured overnight to obtain 80% confluency, pre-treated or not with AmphB at different concentrations (10–20 µg/mL) or MβCD (5 mM) for 30 min and then adsorbed for 1 h at 37 °C with ZIKV at a multiplicity of infection (MOI) of 0.1 or 5 in medium +/− drugs. After adsorption, unbound virus was removed by aspiration and infected cells were cultured in fresh medium +/− drugs for 2 h or 1-2-4 days. In some experiments, AmphB was added only during 1 h of virus adsorption and then aspirated together with the unbound virus. Cell viability was assessed by trypan blue dye exclusion test: cells were incubated for 3 min at room temperature in PBS containing 0.4% Trypan and then counted in the hemacytometer to determine the percentage of cells that have clear cytoplasm (viable cells) versus cells that have blue cytoplasm (non-viable cells).

To analyse the virucidal activity of AmphB, the drug at 10 µg/mL was added to ZIKV stock and incubated at 37 °C for 1 h. Vero cells were then infected with a 100× dilution of the virus–AmphB mixture, corresponding to a final 0.1 MOI, and viral detection was performed on cell supernatants by qRT-PCR after 2 and 4 days of culture.

### 2.3. Immunofluorescence, Image Acquisition and Analysis 

For immunofluorescence staining Vero cells were plated on 8-well chamber slides at a density of 2 × 10^4^ cells/well. AmphB treatments and ZIKV infection were performed as described above. At 24 h post-infection cells, were washed twice with PBS, fixed in 4% paraformaldehyde (20 min, 4 °C), quenched with 50 mM NH_4_Cl in PBS (10 min, RT), permeabilized with PBS containing 0.1% Triton X-100 (10 min, RT), blocked with 3% BSA and 0.05% Tween 20 and then stained with anti-pan-flavivirus antibody (1:400, MAB10216, clone D1-4G2, Millipore, Burlington, Massachussets, USA) followed by donkey anti-mouse Alexa Fluor 594 or AlexaFluor 547 antibodies (both 1:200, Invitrogen, Waltham, Massachussets, USA). Lipid raft clustering was assessed by GM1 ganglioside distribution using the Vybrant™ Alexa Fluor™ 488 Lipid Raft Labeling Kit (#V34403 Molecular Probes, Eugene, Oregon, USA). After infection and drug treatments, live cells were labelled with Alexa Fluor 488 conjugated-CT-B (1 μg/mL, 10 min, 4 °C), followed by anti–CT-B antibody (15 min, 4 °C) to crosslink CT-B. Cells were then fixed and treated as described above. DNA was stained with 0.1 µg/mL 4,6-diamidino-2-phenylindole (DAPI, (Sigma Aldrich, St. Louis, MO, USA) and chambered slides were mounted with the open-well mounting medium (Ibidi GmbH, Grafelfing, Germany)).

Images were acquired as 1600 × 1600 px at the Olympus iX83 FluoView1200 laser scanning confocal microscope using an UPLSAPO20X v.7.8 NA0.75 (Olympus, Tokio, Japan), line average 2, 405 nm and 635 nm lasers. Percentage of infected cells was established from around 10.000 cells for each sample by the Cell Counting tool of MetaMorph software (Molecular Device, San Jose, CA, USA). Stack images were 800 × 800 px acquired at the same microscope using an UPLSAPO60X NA1,35 (Olympus), zoom 2, z-step of 200 nm, 405 nm, 473 nm and 559 nm lasers and then visualized and analysed in 3D by Imaris v8.1.2 software (Bitplane, Oxford Instruments, Abingdon, UK). For 3D volume rendering, Normal Shading Mode was used.

For the 3D analysis, the Surfaces tool was used instead. In particular, for each field, a 3D surface mapping of the lipid raft signal was generated, and the Sum Fluorescent Intensity contained in the 3D structure was quantified.

### 2.4. Viral Detection by Quantitative Reverse Transcription PCR 

In order to assess the effect of AmphB on viral replication, cell supernatant was collected at different time points after infection. A total of 200 µL of supernatant was used for viral RNA extraction using a QIAMP Viral RNA Mini Kit (QIAGEN Hilden, Germany). In order to assess the effect of AmphB on viral entry, RNA was extracted from infected cells two hours after infection. Cells were washed with cold PBS 1× to remove virions attached on the cells surface, scraped, collected with lysis buffer (QIAMP Viral RNA Mini Kit), and homogenized by using QIAshredder spin column (QIAGEN Hilden, Germany). A total of 200 μL of homogenized sample was used for RNA extraction by using QIAMP Viral RNA Mini Kit (QIAGEN Hilden, Germany), as described for the supernatants. Viral titres were determined by quantitative reverse transcription PCR (qRT-PCR). Specific primers ZIKV 1086 (Zvf1086-CCGCTGCCCAACACAAG) and ZIKV 1162c (Zvr1162c-CCACTAACGTTCTTTTGCAGACAT) were used, with 5-FAM as the reporter dye for the probe (ZvP_1107-5FAM-AGCCTACCTTGACAAGCAGTCAGACACTCAA-TAMRA) [13]. Crossing point values were compared with a standard curve obtained from 10-fold serial dilutions of a virus stock of known concentration [13,14], as determined by plaque assay—viral titres were consequently expressed as “plaque forming units equivalents” (PFU eq).

### 2.5. Lipid Rafts Isolation by Sucrose Density Gradient 

Control and ZIKV-infected Vero cells left untreated or treated with AmphB (10 μg/mL) were lysed with MES buffer (25 mM Mes-2-morpholino-ethanesulfonic acid monohydrate at pH 6.5, 0.15 M NaCl) containing 1% (*v*/*v*) of Triton X-100, protease inhibitors (0.1 μg/mL PMSF-Phenylmethylsulfonyl fluoride, 2 μg/mL aprotinin, 2 μg/mL leupeptin, 1 μg/mL pepstatin A) and 1 mM sodium orthovanadate. Cell lysates were homogenized with tight pestles (Wheaton, Milville, NJ, USA) and equal volumes were mixed with 80% sucrose to bring the final density to 40%, placed at the bottom of ultracentrifuge tubes and overlaid with 30% and 5% sucrose solutions. The sucrose gradient was ultracentrifugated in SW60Ti rotor (Beckman Instruments, Palo Alto, CA, USA) at 45,000 rpm for 16 h and then divided in twelve 0.375 mL fractions from the top. Next, 40 μL of the individual fractions were analysed to sodium dodecyl sulfatepolyacrylamide gel electrophoresis (SDS-PAGE) and immunoblotting. Raft fractions 4–6 were distinguishable as a floating opaque ring migrating at 20% sucrose. Fractions 1–2 did not contain proteins; fraction 12 represented the nuclear portion and, thus, was not subjected to SDS-PAGE. 

### 2.6. Immunoblotting 

Samples were resuspended in an appropriate volume of sample buffer (100 mM TRIS/HCl pH 6.8, 1% SDS, 10% glycerol, 1% dithiothreitol and blue bromophenol) and 50 μM DTT and boiled for 5 min at 100 °C. Proteins were separated on an SDS PAGE gel and then transferred on nitrocellulose membrane by Western blotting. The membrane was blocked with 5% non-fat dry milk in TBST buffer (10 mm Tris-HCl (pH 8.0), 150 mm NaCl, 0.1% Tween 20) for 45 min at RT, followed by incubation for 1 h at RT with primary antibodies: mouse anti-Flotillin-1 (BD Transduction Laboratories, Franklin Lakes, NJ, USA), mouse anti-TFR-1 (Santa Cruz Biotechnology, Dallas, TX, USA). Then, the filter was incubated with the appropriate horseradish peroxidase-conjugated secondary antibody (Bio-Rad Hercules, CA, USA)) for 1 h at RT and the reactivity was detected by the enhanced chemiluminescence kit (Pierce Biotechnology, Waltham, MA, USA). The fold-enrichment of the proteins was determined by densitometric quantitation using ImageJ version 1.53t software.

### 2.7. Statistical Analysis 

Statistical analysis was performed using the Mann–Whitney U test using GraphPad Prism, version 7.0 software (GraphPad Software, San Diego, CA, USA). A *p* value < 0.05 was considered statistically significant.

## 3. Results

### 3.1. Disruption of Lipid Raft Architecture by AmphB Inhibits ZIKV Replication and Infection of Vero Cells

**Figure 1:**
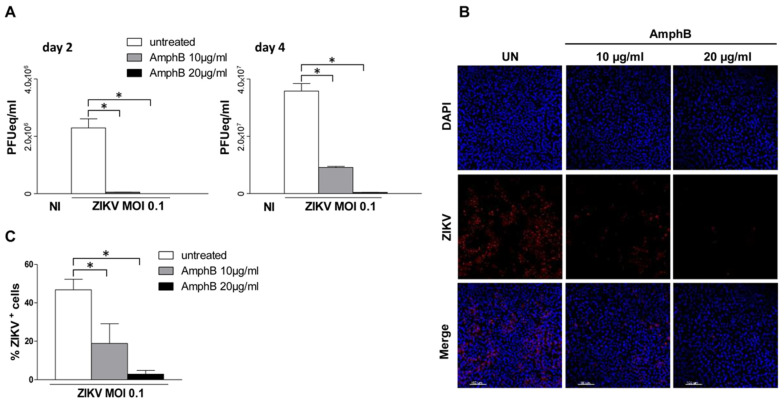
Disruption of lipid raft architecture by AmphB inhibits ZIKV replication and infection of Vero cells. Vero cells were left untreated or pre-treated with increasing doses of AmphB (10–20 µg/mL) for 30 min and adsorbed with ZIKV at MOI of 0.1 in medium +/− AmphB for 1 h at 37 °C. NI: non-infected. (**A**) After 2 and 4 days of culture in fresh medium +/−, AmphB viral titre was determined by qRT-PC and expressed as PFU equivalents/mL (PFUeq/mL). Drug treatment significantly inhibited viral replication in a dose-dependent manner. (**B**) Immunofluorescence analysis after 24 h of culture in fresh medium +/− AmphB. Cells were fixed and stained with anti-pan-flavivirus antibody (ZIKV) followed by donkey anti-mouse AlexaFluo 647 antibody (red). Nuclei were stained with DAPI (blue). Scale bar is 100 µm. Images are from one representative experiment out of three. (**C**) Percentage of infected cells was established for each sample using the Cell Counting tool of MetaMorph software. Data showed a dose-dependent inhibitory effect of the drug on cell infection that decreased from 47% to 19% at 10 µg/mL and 20 µg/mL, respectively. Results are expressed as mean ± standard deviation (SD) of three independent experiments performed. Significance was determined by GraphPad Prism using the nonparametric Mann–Whitney U test. * *p* value < 0.05 was considered statistically significant.

We first evaluated the effect of AmphB on ZIKV infection of Vero cells, a polyene antibiotic drug which we have previously shown to be a potent lipid raft disrupting agent by sequestering membrane cholesterol which is the glue that maintains lipid raft architecture [15]. Vero cells were pre-treated or not with increasing doses of AmphB (10–20 µg/mL) or of 5 mM MβCD, the classical lipid raft disrupting agent, for 30 min at 37 °C. Cells were then adsorbed with ZIKV at MOI of 0.1 in medium +/− drugs for 1 h at 37 °C. After 2 and 4 days of culture in fresh medium +/− drugs, viral detection on cell supernatants was performed by quantitative reverse transcription PCR (qRT-PCR). AmphB treatment at both concentrations did not significantly alter cell viability after 1, 2 and 4 days of culture (Figure S1). As shown in Figure 1A, AmphB strongly inhibited viral replication in a dose-dependent manner at day 2 and 4 of culture as well as MβCD at day 1. (Figure S2). Immunofluorescence analysis at 24 h post-infection also showed a dose-dependent inhibitory effect of AmphB on cell infection that decreased from 47% to 19% and 3% at 10 µg/mL and 20 µg/mL, respectively (Figure 1B,C), suggesting the involvement of lipid rafts in ZIKV cell infection.

### 3.2. ZIKV Exploits Lipid Rafts to Enter and Infect Vero Cells

**Figure 2:**
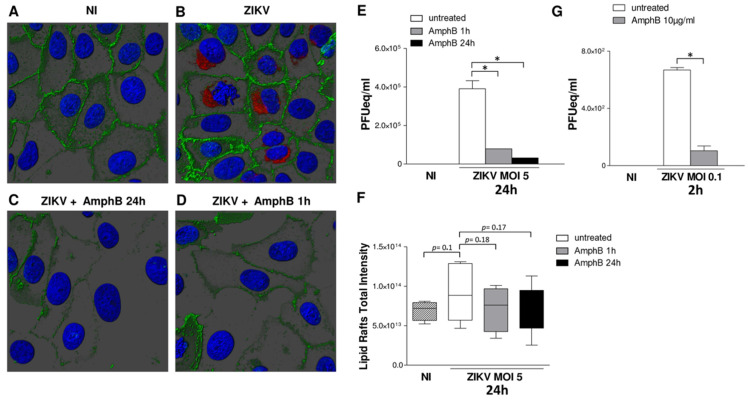
ZIKV exploits lipid rafts to enter and infect Vero cells. Vero cells were adsorbed with ZIKV at MOI 5 for 1 h at 37 °C in medium +/− AmphB (10 µg/mL). Unbound virus was removed, and infected cells were cultured in fresh medium +/− AmphB for 24 h or the drug was added only during 1 h of virus adsorption. At 24 h post-infection, cells were stained with the lipid raft marker CTB (green), fixed and stained with anti-pan-flavivirus antibody (ZIKV) followed by donkey anti-mouse Alexa Fluor 594 antibody (red). Nuclei were stained with DAPI (blue). 3D images were visualized and analysed by Imaris v8.1.2 software. NI: non-infected. (**A**,**B**) As compared to NI cells, ZIKV infection induced a lipid raft reorganization on cell surface. (**C**) Treatment with AmphB for 24 h disrupted lipid raft architecture and inhibited cell infection. (**D**) Treatment with AmphB only along 1 h of ZIKV adsorption disrupted lipid raft architecture and impaired the first steps of viral entry. Images are shown from one representative experiment out of three. (**E**) Viral titre was determined by qRT-PCR and expressed as PFU equivalents/mL (PFUeq/mL). Both drug treatments significantly inhibited viral replication. (**F**) Quantification of lipid rafts total intensity by 3D image analysis. The 3D surface enclosing the lipid raft signal was generated using the same parameters for all the images. The Sum Fluorescent Intensity inside the volume was measured and results summarized in the graph. ZIKV infection increased the lipid rafts fluorescence intensity, whereas both drug treatment decreased this value. (**G**) Treatment of AmphB during the first two hours of ZIKV infection (MOI 5) reduced viral entry as measured by titration of viral RNA extracted from infected cells and expressed as PFU equivalents/mL (PFUeq/mL). Results are expressed as mean ± SD of three independent experiments performed. Significance was determined by the nonparametric Mann–Whitney U test using GraphPad Prism. * *p* value < 0.05 was considered statistically significant.

To confirm these data, Vero cells infected with ZIKV at MOI 5 were stained with the lipid raft marker cholera toxin B which binds to ganglioside GM1 and analysed by confocal microscopy 24 h post-infection. As shown in Figure 2A,B, ZIKV infection induced a lipid raft reorganization on Vero cells surface as compared to uninfected cells. Treatment with AmphB during 24 h of infection disrupted lipid raft architecture and cell infection (Figure 2C) and significantly inhibited viral replication (Figure 2E), as assessed by viral titration by real-time RT PCR on cell supernatant, after 24 h infection. In order to investigate the step of infection affected by AmphB, the drug was added to infected cells and removed 1 h post-adsorption. This treatment also inhibited lipid raft reorganization and cell infection (Figure 2D) and viral replication (Figure 2E), suggesting that lipid rafts may be involved in the first steps of viral entry. To quantify lipid raft reorganization, we evaluated the total fluorescence intensity by 3D images analysis; although not statistically significant, an increase in lipid rafts fluorescence intensity following ZIKV infection was observed, whereas both treatments with AmphB decreased this value (Figure 2F).

To confirm lipid raft involvement in the first steps of viral entry, we also performed titration of viral RNA extracted from infected cells, pre-treated or not with 20 µg/mL of AmphB and collected after 2 h from infection with ZIKV (MOI 5) in medium +/− drug. As shown in Figure 2G, AmphB significantly reduced ZIKV entry in Vero cells. 

### 3.3. ZIKV Infection Induces an Enrichment of Flotillin-1 into Raft Fractions Which Is Inhibited by AmphB Treatment

**Figure 3:**
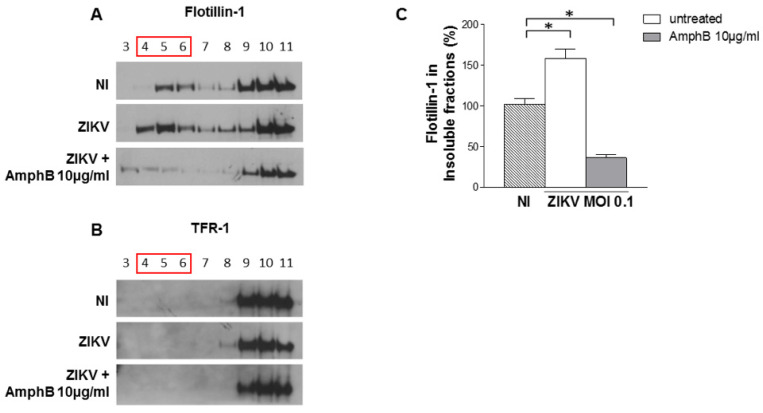
ZIKV infection induces an enrichment of flotillin-1 into raft fractions which is inhibited by AmphB treatment. Control and infected cell lysates +/− AmphB (10 µg/mL) were solubilized with 1% Triton X-100 at 4 °C and separated into membrane and cytosolic fractions by sucrose gradient centrifugation. Samples from each fraction were analysed by Western blotting using the lipid raft marker flotillin-1 and the non-raft marker TFR-1. NI: non-infected. (**A**) The localization of flotillin-1 in infected cells increased in raft fractions 4 and 5, whereas AmphB treatment induced a loss of flotillin-1 in the same fractions. (**B**) ZIKV infection and AmphB did not affect lipid raft localization of TFR-1. One representative experiment out of three performed is shown. (**C**) Densitometric quantification analysis of flotillin-1 (4–6) by using ImageJ software. Results are expressed as mean ± SD of three independent experiments performed. Significance was determined by the nonparametric Mann–Whitney U test using GraphPad Prism. * *p* value < 0.05 was considered statistically significant.

Control and infected cell lysates were prepared in ice cold lysis buffer containing 1% Triton X-100, then separated into membrane and cytosolic fractions by sucrose gradient centrifugation; samples from each fraction were analysed by Western blotting using the lipid raft marker flotillin-1 and the non-raft marker transferrin receptor (TFR-1). As shown in Figure 3A, flotillin localization increased in 4 and 5 raft fractions, whereas TFR-1 was not modified (Figure 3B). The treatment of infected cells with AmphB induced a loss of flotillin-1 in raft fractions but did not alter TFR-1 localization (Figure 3A,B). A densitometric quantification analysis of flotillin-1 (Figure 3C) confirmed the reorganization of lipid raft architecture and lipid raft perturbation by the drug upon infection.

### 3.4. AmphB Does Not Have Virucidal Activity on ZIKV

**Figure 4:**
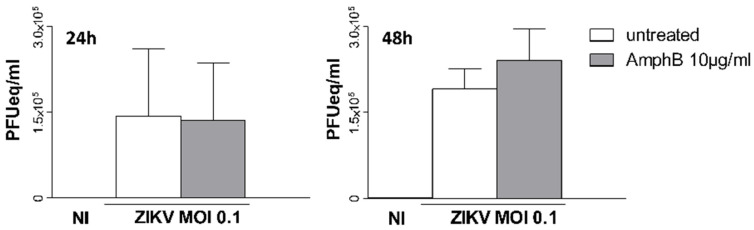
AmphB does not have virucidal activity on ZIKV. AmphB at 10 µg/mL was added to ZIKV stock and incubated at 37 °C for 1 h. Vero cells were then infected at MOI 0.1 with AmphB-treated virus; viral detection was performed on cell supernatants by qRT-PCR after 2 and 4 days of culture and expressed as PFU equivalents/mL (PFUeq/mL). NI: non-infected. Results are expressed as mean ± SD of two independent experiments performed.

Finally, we asked whether AmphB would have a direct virucidal activity on ZIKV. AmphB (10 µg/mL) was added to ZIKV stock, and the stock was incubated at 37 °C for 1 h. Vero cells were then infected at MOI 0.1 and viral detection was performed on cell supernatants by qRT-PCR after 2 and 4 days of culture. As shown in Figure 4, AmphB did not inhibit viral replication on both days 2 and 4 of culture, excluding the virucidal activity of the drug that could alter the stability of ZIKV virions.

## 4. Conclusions

In this study, we show for the first time that ZIKV employs cell lipid rafts as a portal of entry into host cells. The identification of a new route for viral entry into host cells introduces a potential new tool for designing additional therapeutic intervention in the viral disease arena. Targeting lipid rafts as a strategy against infection has already been proposed for other viruses. Drugs such as filipin, nystatin, saponin, and MβCD cause disruption to the lipid rafts by directly depleting cholesterol from host cell plasma membrane and, consequently, they have been proposed as “antiviral” agents [14]. Here, we show that also the cholesterol-binding AmphB by the same mechanism disrupts lipid raft architecture and inhibits both ZIKV entry and replication. The AmphB deoxycholate formulation is known to have a high-dose-limiting toxicity, but now less toxic formulations are available, consisting of liposomes with AmphB intercalated within the membrane [16]. LipAmph is already used in several clinical disorders, such as febrile neutropenia, or infections, such as systemic aspergillosis, candidiasis and visceral leishmaniasis [17]. Currently, no specific antiviral drugs or preventive vaccines are available for ZIKV infection. Altogether, our in vitro results encourage further studies on drug repurposing for ZIKV infection of this already approved polyene antibiotic. 

## Data Availability

Not applicable.

## Supplementary Materials

The following supporting information can be downloaded at: https://www.mdpi.com/article/10.3390/v14092059/s1, Figure S1: Cell viability analysis after AmphB treatment; Figure S2: MβCD inhibits ZIKV replication in Vero cells.
