# Supplementary material for: Zika Virus Exploits Lipid Rafts to Infect Host Cells
Source: Viruses. 2022 Sep 16;14(9):2059. doi: 10.3390/v14092059 (PMC9506595; doi:10.3390/v14092059)
Supplement: Supplementary file 1 [file viruses-14-02059-s001.zip › viruses-1881575-supplementary.pdf]

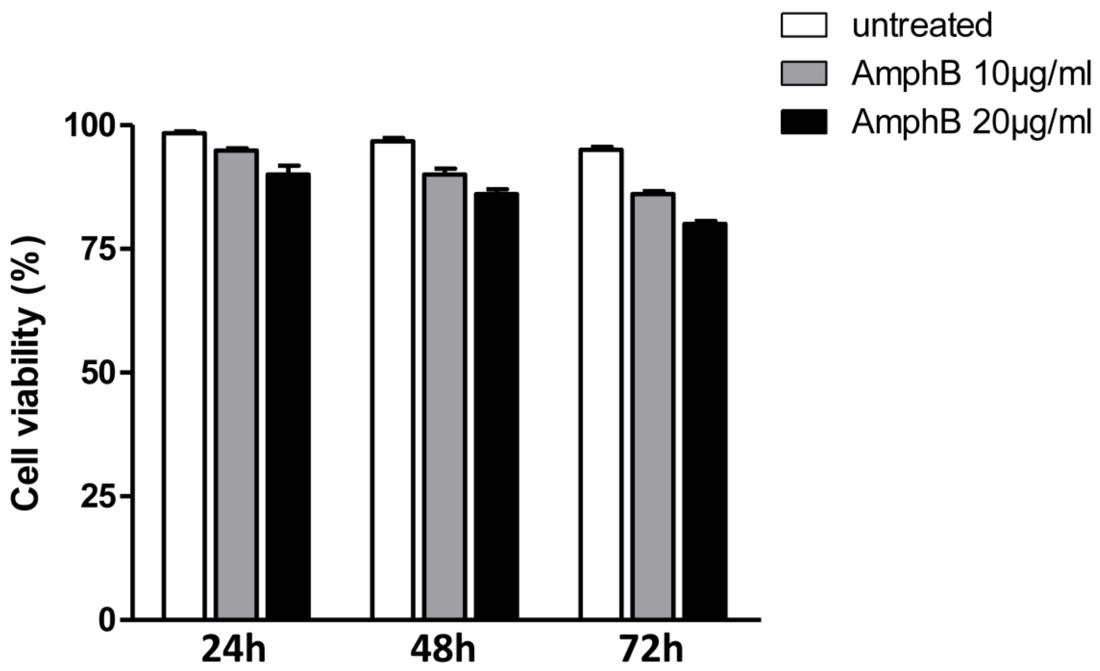

Figure S1. Cell viability analysis after AmphB treatment.

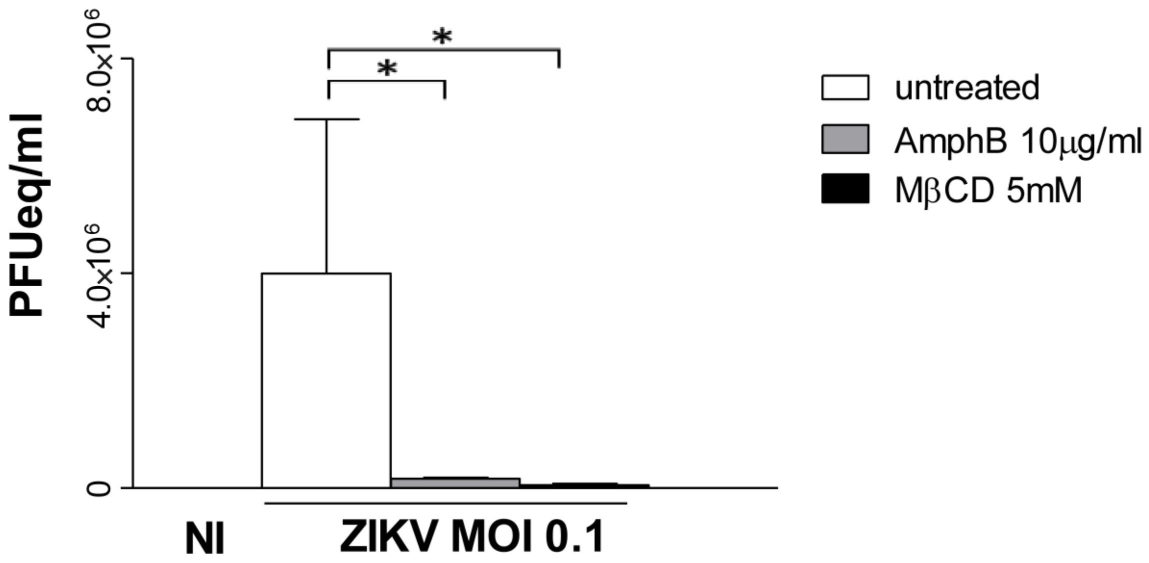

Figure S2. MβCD inhibits ZIKV replication in Vero cells. * p value < 0.05.
